# Biochar-Mediated Control of Phytophthora Blight of Pepper Is Closely Related to the Improvement of the Rhizosphere Fungal Community

**DOI:** 10.3389/fmicb.2020.01427

**Published:** 2020-07-08

**Authors:** Guangfei Wang, Yan Ma, Hafizah Yousuf Chenia, Roshini Govinden, Jia Luo, Gaidi Ren

**Affiliations:** ^1^Institute of Agricultural Resources and Environments, Jiangsu Academy of Agricultural Sciences, Nanjing, China; ^2^Key Laboratory of Agro-Environment in Downstream of Yangtze Plain, Ministry of Agriculture and Rural Affairs, Nanjing, China; ^3^Discipline of Microbiology, School of Life Sciences, College of Agriculture, Engineering and Science, University of KwaZulu-Natal, Durban, South Africa

**Keywords:** biochar, application time, Phytophthora blight of pepper, soil chemical properties, fungal community, biocontrol fungi

## Abstract

Biochar is a new eco-material with the potential to control soilborne diseases. This study explored the relationship between the rhizosphere fungal community and the suppression of Phytophthora blight of pepper in the context of time after biochar application. A pot experiment was conducted and rhizosphere soils were sampled to determine the biochar-induced soil chemical properties, fungal community composition, and abundance of biocontrol fungi. The biochar-enriched fungal strains were screened by the selective isolation method, and their control effects against Phytophthora blight of pepper were determined using a pot experiment. Biochar treatments effectively inhibited pathogen growth and controlled the disease, with biochar applied immediately before planting (BC0) having greater effects than that applied 20 days before planting (BC20). Compared to the control, biochar-amended rhizosphere soils had a higher pH, available nutrient content, and fungal richness and diversity. Moreover, biochar treatments significantly increased the abundance of potential biocontrol fungi. The proliferation in BC0 was stronger as compared to that in BC20. Several strains belonging to *Aspergillus*, *Chaetomium*, and *Trichoderma*, which were enriched by biochar amendment, demonstrated effective control of Phytophthora blight of pepper. Canonical correspondence and Pearson’s correlation analysis showed that a high content of soil-available nutrients in biochar treatments was favorable to the proliferation of beneficial fungi, which was negatively correlated with both the abundance of *Phytophthora capsici* and disease severity. In conclusion, biochar-mediated improvement in the fungal community suppressed the Phytophthora blight of pepper. The biochar application time had a great impact on the control effect, possibly due to the short-term proliferative effect of the biochar on biocontrol fungi.

## Introduction

Biochar, the solid by-product of biomass pyrolysis, features stable aromatic carbon structures, large surface areas, and high contents of certain nutrients and organic carbon (Sohi et al., 2010; Lehmann et al., 2011). In addition to sequestering carbon and reducing greenhouse gas emissions, biochar application can improve the soil pH, moisture retention, physical structure, nutrient status, and biological properties, which in turn enhance plant growth and health under biotic and abiotic stress conditions (Sohi et al., 2010; Egamberdieva et al., 2016; Nair et al., 2017; Meng et al., 2019).

Recent studies have shown that biochar application can effectively control soilborne plant diseases caused by pathogenic fungi and bacteria, such as *Fusarium oxysporum*, *Rhizoctonia solani*, and *Ralstonia solanacearum* (Jaiswal et al., 2014, 2015; Elmer, 2016; Zhang et al., 2017; Gao et al., 2019; Chen et al., 2020). Our previous study first reported that the addition of biochar to soil resulted in a good control effect of Phytophthora blight of pepper, caused by the pathogenic oomycete *Phytophthora capsici* L. (Wang et al., 2017). However, the mechanisms remain unknown. Biochar-induced soil chemical properties are closely associated with the control of diseases caused by soilborne bacteria (Zhang et al., 2017; Gao et al., 2019; Chen et al., 2020), but whether it is conducive to the control of diseases caused by oomycetes needs to be explored.

Biochar has a high C/N ratio and can provide a habitat that is conducive to colonization by fungal hyphae (Lehmann et al., 2011). In addition, many fungi can proliferate by degrading the organic components within biochar (Anyika et al., 2015). Therefore, biochar application is beneficial for soil fungal growth. This has been confirmed by a significant increase in fungal abundance and changes in fungal communities following biochar amendment (Bamminger et al., 2014; Yao et al., 2017; Zhang et al., 2019; Wu et al., 2020). In addition, several researchers observed the enrichment of potential biocontrol fungi, such as *Trichoderma* and *Paecilomyces*, in biochar-amended soil (Hu et al., 2014; Elmer, 2016; Zhang et al., 2019). Thus, we hypothesized that biochar-mediated improvement of the rhizosphere fungal community, especially the enrichment of biocontrol fungi, is closely related to the suppression of soilborne diseases. Until now, no in-depth studies have analyzed the association between biochar-mediated disease control and the fungal community. Jaiswal et al. (2017, 2018) determined the fungal and bacterial communities in response to biochar and established a relationship between the bacterial community and disease suppression, but did not establish a relationship between the fungal community and disease suppression.

There are many studies showing the influence of feedstock, pyrolysis temperature, and application dose of biochar on their disease control effects (Jaiswal et al., 2014, 2015; Lu et al., 2016; Wang et al., 2017; Chen et al., 2020), but not one focused on the biochar application time. The application of biochar just before planting may maximize the control effect because of the short-term effect of biochar on soil biological properties (Farrell et al., 2013; Jiang et al., 2016). However, application a few days before planting to form disease-suppressing soil microflora may help improve the control effect. Therefore, we speculate that the biochar application time has a significant influence on its control effect and that this is caused by the time-dependent influence of the biochar on the soil microbial community.

Our aims for this study were as follows: (i) to analyze whether the biochar-mediated control of Phytophthora blight of pepper is related to an improved fungal community; (ii) to study whether the response of soil chemical properties to biochar amendment contributes to the improvement of the rhizosphere fungal community and suppression of Phytophthora blight of pepper; and (iii) to explore the association between the biochar application time and the rhizosphere fungal community as well as disease suppression. Therefore, we investigated the function of biochar in shaping soil chemical properties, fungal community composition, abundance of biocontrol fungi, pathogen abundance, and disease severity under different application times. Moreover, the screening and verification of biocontrol agents were performed to ascertain the relationship between biochar-enriched biocontrol fungi and disease suppression by biochar. The results of our work are expected to provide a practical approach for the utilization of biochar to control soilborne diseases and provide theoretical support for the further enhancement of plant disease control with biochar application.

## Materials and Methods

### Soil and Biochar Preparation

The experimental soil had a sandy loam texture. It was collected from the top 20-cm layer in a pepper greenhouse in Huangma town, Jiangsu Province, China. The basic chemical characteristics of the soil were as follows: pH, 7.44; electrical conductivity (EC), 1,879 μs/cm; total N, 3.1 g/kg; total P, 2.0 g/kg; total K, 12.6 g/kg; and organic matter, 29.4 g/kg.

The biochar was made from corn stalk based on the methods of Xie et al. (2013) and was passed through a 40-mesh screen. The basic characteristics of the biochar were as follows: pH, 9.73; EC, 5,763 μs/cm; total C, 490 g/kg; total H, 23 g/kg; total O, 146 g/kg; total N, 17.5 g/kg; ash, 324 g/kg; organic matter, 286 g/kg; available P, 2.2 g/kg; and available K, 24.7 g/kg.

### Pot Experiment

Three treatments were established: (i) soil incubated for 20 days without biochar (CK); (ii) CK soil amended with biochar at a rate of 13.3 g/kg just before planting (BC0); and (iii) CK soil amended with biochar at a rate of 13.3 g/kg 20 days before planting (BC20). Treatments were prepared in triplicate and incubated at ambient temperatures of 15–30°C. The soil moisture content was maintained approx. 20% during incubation. All of the treatments were mixed with a *P. capsici* zoospore suspension at a density of 100 zoospores per gram soil at the end of the incubation period. Each treatment was repeated three times, and each replicate had 20 pots (12 cm × 15 cm, diameter × height). Each pot contained 600 g of soil and one 5-week-old pepper plant. The pots were randomly arranged and incubated inside the greenhouse for 45 days, as mentioned above.

The disease index was recorded every 15 days using a 0–4 scale according to Wang et al. (2019). Disease index = [Σ(number of infected plants of a specific scale × the specific scale/(4 × (total number of plants))] × 100%.

### Sample Collection and DNA Extraction

Before planting, the soil samples were harvested from all of the replicates. At 15, 30, and 45 days after planting, five plants were randomly dug out from each replicate and the rhizosphere soil was harvested. The soil samples were passed through a 10-mesh screen. One portion of each sample was air dried and then stored at 4°C for the later analysis of pH, EC, organic matter, available P, and available K; the other portion was stored at −70°C and used for the later extraction of total DNA and the determination of nitrate N. In addition, the soil samples harvested at day 30 after planting were used for the screening of biochar-enriched biocontrol fungi and the determination of the fungal community composition. Soil total DNA was isolated using a FastDNA SPIN Kit (MP Biomedicals).

### Analysis of Soil Chemical Properties

Soil pH and EC (soil/water = 1:5, *w*/*v*) were measured using a pH meter (Mettler-Toledo FE20, Mettler-Toledo Instrument Factory, Shanghai, China) and an EC meter (DDS307, Shanghai Jingke Instrument Factory, Shanghai, China), respectively. Organic matter, nitrate N, available P, and available K were assayed according to the methods of Wang et al. (2014).

### Quantitative PCR

SYBR Premix Ex Taq (Tli RNaseH Plus) was used to amplify the fungi, *Chaetomium globosum*, *P. capsici*, and *Trichoderma* on a 7500 quantitative PCR instrument (Applied Biosystems, United States). The fungal 18S rRNA gene was quantified using the primer pair NS1-F/FungR (Wang et al., 2014). The number of *C. globosum*, *P. capsici*, and *Trichoderma* were determined with the genus-specific primer pairs SCCgQF/SCCgQR (Aggarwal et al., 2014), CAPFW/CAPRV1 (Wang et al., 2014), and uTf/uTr (Hagn et al., 2007), respectively. TaqMan quantitative PCR (qPCR) of *Aspergillus* and *Penicillium* was conducted using Premix Ex Taq (Probe qPCR), and the primers/probes for *Aspergillus* and *Penicillium* were Asp2/AspG/Probe137 (Goebes et al., 2007) and ITSPF/ITSPR/ProbePENP (Suanthie et al., 2009), respectively. The copy numbers of the target genes in total fungi and *P. capsici* were calculated based on a plasmid-generated standard curve. The standard curves for the calculation of *C. globosum*, *Aspergillus*, *Penicillium*, and *Trichoderma* were prepared with 10-fold serial dilutions of genomic DNA.

### Illumina MiSeq Sequencing and Analysis

Thirty days after planting, rhizosphere soil DNAs were extracted and then subjected to Illumina MiSeq sequencing by Majorbio Co., Ltd. (Shanghai, China). The primers ITS1F (5′-CTT GGT CAT TTA GAG GAA GTA A-3′) and ITS2R (5′-GCT GCG TTC TTC ATC GAT GC-3′) were used for the PCR assay of the fungal internal transcribed spacer (ITS) region of the target ribosomal gene (Bokulich and Mills, 2013). The PCR products were sequenced using the MiSeq sequencing platform, and all sequences were clustered into operational taxonomic units (OTUs) at 97% identity using UPARSE (Kõljalg et al., 2013). The fungal community was characterized in terms of the number of OTUs, Shannon index, coverage, and the richness estimators Chao1 and ACE (abundance-based coverage estimation) using the mothur software (Schloss et al., 2009). OTUs were classified using the UNITE ITS database (Kõljalg et al., 2013). Principal coordinate analysis (PCoA) based on the weighted UniFrac distances was performed to determine the differences in the fungal community composition. Canonical correspondence analysis (CCA) was performed to determine the relationships between the soil chemical properties and the fungal community composition.

### Screening and Assessment of Biochar-Enriched Biocontrol Fungi

Rhizosphere soil samples for the CK and BC0 treatments at 30 days after planting were collected as described above. Ten grams of the soil sample was added to 90 ml 0.85% saline solution and shaken for 30 min at 160 rpm. After performing 10-fold dilutions, 100 μl of the diluted suspensions (10^–2^ and 10^–3^) was plated on potato dextrose agar (PDA) containing 30 mg L^–1^ rifampin in 15 replicates per dilution and incubated at 26°C for 3–7 days. When fungal colonies appeared on the PDA plates, *Trichoderma*, *Chaetomium*, *Penicillium*, and *Aspergillus* spp. strains induced by the BC0 treatment were picked and inoculated onto new PDA plates to obtain pure strains. Morphological features such as colony, mycelium, and spore characteristics were used to avoid replication of the strains. The remaining strains were identified by amplification and sequencing of the respective ITS regions. The nucleotide sequences were analyzed using a similarity search against the GenBank database (Zheng et al., 2011).

The strains were cultivated using potato dextrose broth and then their mycelia were harvested by filtration through sterile gauze. Homogeneous suspensions of propagules were obtained by washing the mycelia with sterile distilled water and homogenizing the washed mycelia with a homogenizer. The experimental soil was mixed with a *P. capsici* zoospore suspension at a density of 100 zoospores per gram soil. The inoculated soil was divided into fungal treatment groups and one control. The fungal inoculation rate was 10 g fresh mycelia/kg soil. The soil inoculated with the pathogen only was set as the control. Each treatment was replicated three times, and there were 15 pepper plants for each replicate. One 5-week-old pepper plant was grown in a pot containing 600 g of soil. All plants were acclimated in the greenhouse for 45 days, as mentioned above. The disease severity was determined at 15 and 30 days after planting. The disease index was assessed as described previously. The biocontrol efficacy was assessed using the following equation: Biocontrol efficacy = [(disease index under control − disease index under fungal treatment)/disease index under control] × 100%.

### Data Analysis

All data were analyzed with the SPSS 19.0 software package (IBM, United States). Statistical significance between the treatments was tested with one-way analysis of variance (ANOVA). *p* < 0.05 was considered statistically significant.

## Results

### Effect on Disease Severity and Pathogen Abundance

**FIGURE 1:**
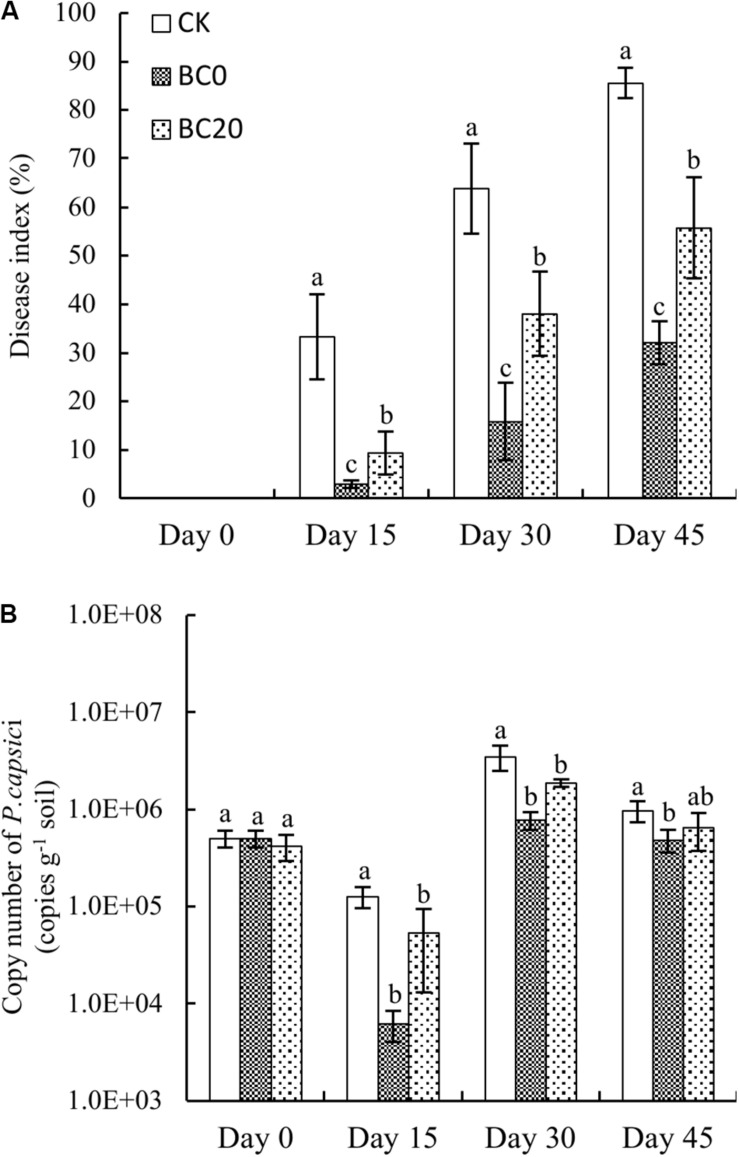
Temporal effects of biochar applied at different times on the disease index **(A)** and abundance of *Phytophthora capsici* **(B)** during the growing period. *Bars* represent average values ± standard error. *Different letters above columns* indicate significant differences (*p* < 0.05) according to one-way ANOVA (*n* = 3).

As shown in Figure 1A, the disease indices of all treatments continued to increase with longer growing period. Biochar amendment significantly alleviated the disease development rate and disease indices compared to CK. In addition, the disease index of BC0 was reduced compared to that of BC20. The disease indices of BC0 and BC20 on day 15 were reduced by 91 and 72% compared to those of CK, and those on day 45 were reduced by 63 and 35%, respectively. This suggested that biochar amendment reduced disease development and that the control effect of BC0 was stronger than that of BC20.

The pathogen abundance in each treatment decreased by day 15 and then increased by day 30, followed by a gradual decrease on day 45 (Figure 1B). The pathogen abundance under the biochar treatments was significantly reduced relative to that under CK, and that under BC0 was significantly reduced compared to that under BC20 during the entire growing period. In comparison, pathogen abundance under BC0 and BC20 on day 15 after planting was reduced by 95 and 58%, respectively, and by 50 and 34%, respectively, on day 45 after planting. Notably, biochar-induced suppression of *P. capsici* diminished over time.

### Effect on Soil Chemical Properties and Fungal Communities After Incubation for 20 Days

#### Chemical Properties

The soil chemical properties changed to varying degrees after amendment with biochar and incubation for 20 days (Supplementary Table S1). Biochar amendment markedly increased the EC and contents of organic matter, available P, and available K, which were correlated with the high contents of ash, organic matter, available P, and available K in the biochar.

#### Fungal Abundance and Community Composition

**FIGURE 2:**
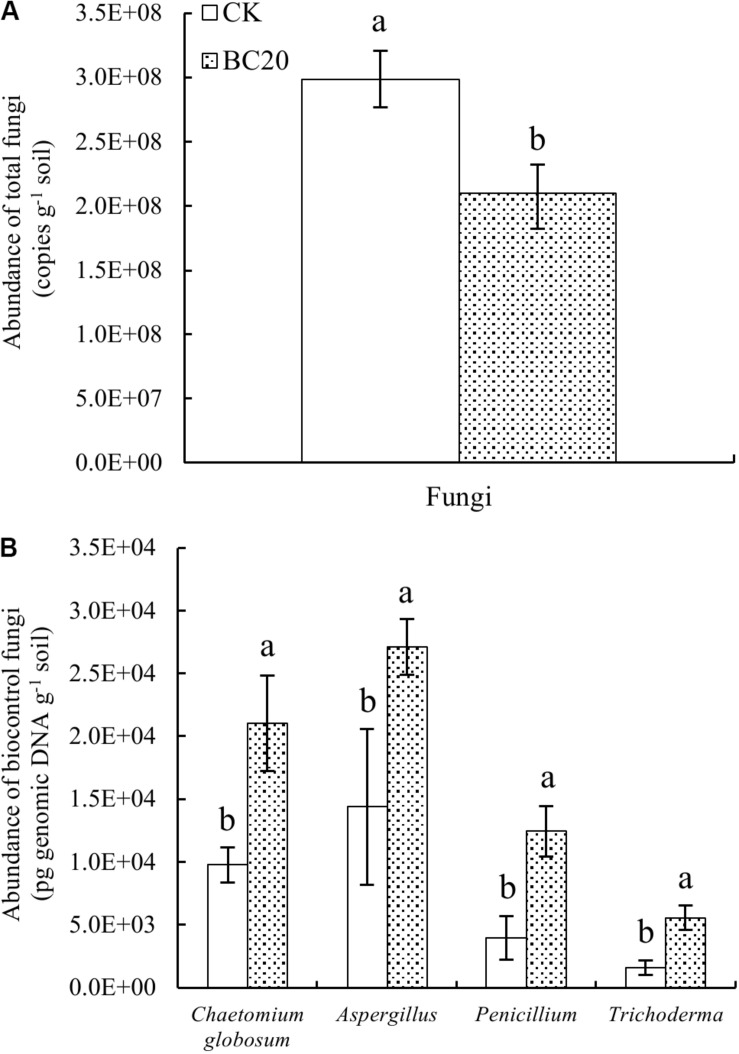
Effect of biochar amendment on the abundance of total fungi **(A)** and potential rhizosphere-associated biocontrol fungi **(B)** after incubation for 20 days. *Bars* represent the standard error of each mean. *Different letters above columns* indicate significant differences (*p* < 0.05) according to one-way ANOVA (*n* = 3).

The qPCR results suggested a significantly reduced abundance of total fungi and a significantly increased abundance of *C. globosum*, *Aspergillus*, *Penicillium*, and *Trichoderma* 20 days after the amendment of the soil with biochar (Figures 2A,B). In addition, the results of high-throughput sequencing indicated a reduced relative abundance of *Cladosporium* and *Emericella*. Furthermore, biochar significantly increased the relative abundance of *Chaetomium*, *Funneliformis*, *Penicillium*, and *Trichoderma* (Supplementary Figure S1). Therefore, although biochar amendment significantly reduced the total fungal abundance after 20 days of incubation, it increased the abundance of potential biocontrol fungi.

### Effect of on Soil Chemical Properties and Fungal Community During Planting

#### Chemical Properties

**FIGURE 3:**
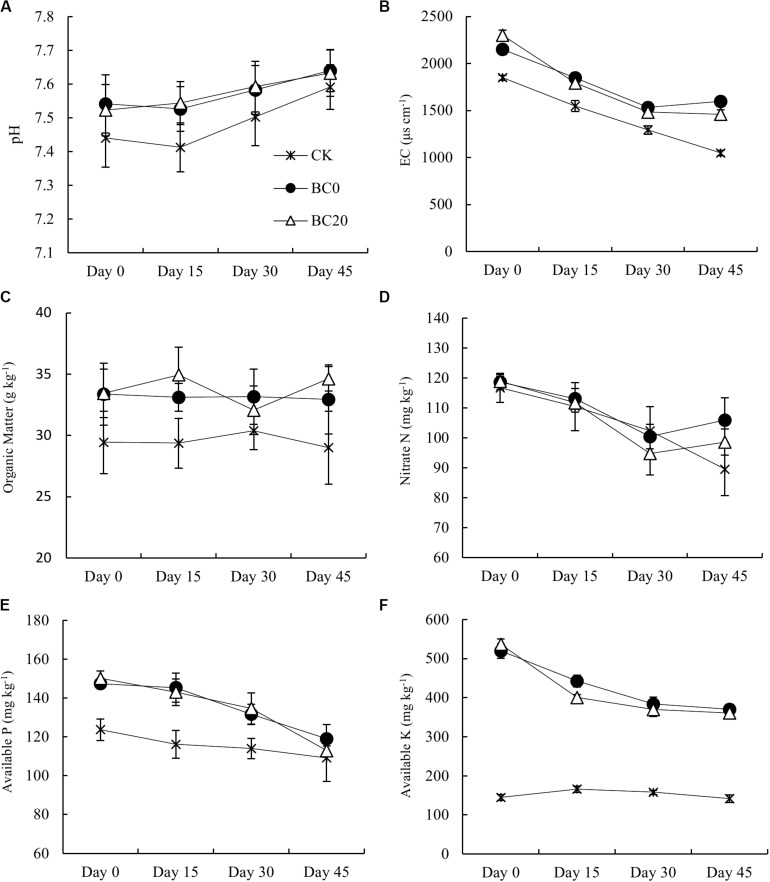
Temporal effects of biochar applied at different times on soil chemical properties during the growing period. Soil chemical properties included pH **(A)**, electrical conductivity **(B)**, organic matter **(C)**, nitrate nitrogen **(D)**, available P **(E)**, and available K **(F)**. *Bars* represent average values ± standard errors (*n* = 3).

Both biochar treatments significantly increased the soil pH, EC, and contents of organic matter, available P, and available K compared with CK during the entire growing period (Figure 3). The difference between biochar treatments, however, was not significant. The promotion of pH, available P, and available K by biochar amendment gradually declined with extended planting time. Compared to CK, BC0 and BC20 had 1.36 and 1.11% higher pH values before planting, which decreased by 0.64 and 0.54% at 45 days after planting, respectively. Similarly, the available P content increased significantly by 19.13 and 21.38% before planting in BC0 and BC20, but at 45 days after planting, the differences were only 9.02 and 3.40%, respectively. The available K contents were 259 and 271% higher in BC0 and BC20 before planting and were still 161 and 155% higher than CK at 45 days after planting, respectively. Thus, biochar-induced changes in soil nutrient qualities and chemical properties were only slightly affected by the time after biochar application, but more obviously affected by the planting time.

#### Fungal Richness and Diversity

The community richness indices, OTUs, Chao1, and ACE values were higher under the biochar treatments than under CK, and significant differences in the OTUs and Chao1 values were observed at 30 days after planting (Table 1). The Shannon diversity indices of the biochar treatments were higher than those of the CK, but significant difference was not observed. No significant difference in the richness and diversity indices was observed between BC0 and BC20.

**TABLE 1:** Fungal richness and diversity indices at 30 days after transplanting.

	OTUs	ACE	Chao1	Coverage	Shannon
CK	235 ± 19b	456 ± 98b	372 ± 70b	0.962 ± 0.009a	3.36 ± 0.27a
BC0	297 ± 12a	595 ± 59a	492 ± 27a	0.951 ± 0.006a	3.60 ± 0.15a
BC20	284 ± 24a	649 ± 137a	466 ± 59ab	0.950 ± 0.013a	3.75 ± 0.26a

*OTUs, operational taxonomic units; ACE, abundance-based coverage estimation; Chao1, Chao estimator of microbial richness; Coverage, Good’s coverage index; Shannon, Shannon diversity index. Different letters after the values in the same column indicate significant differences (p < 0.05) according to one-way ANOVA (n = 3).*

#### Fungal Community Composition

**FIGURE 4:**
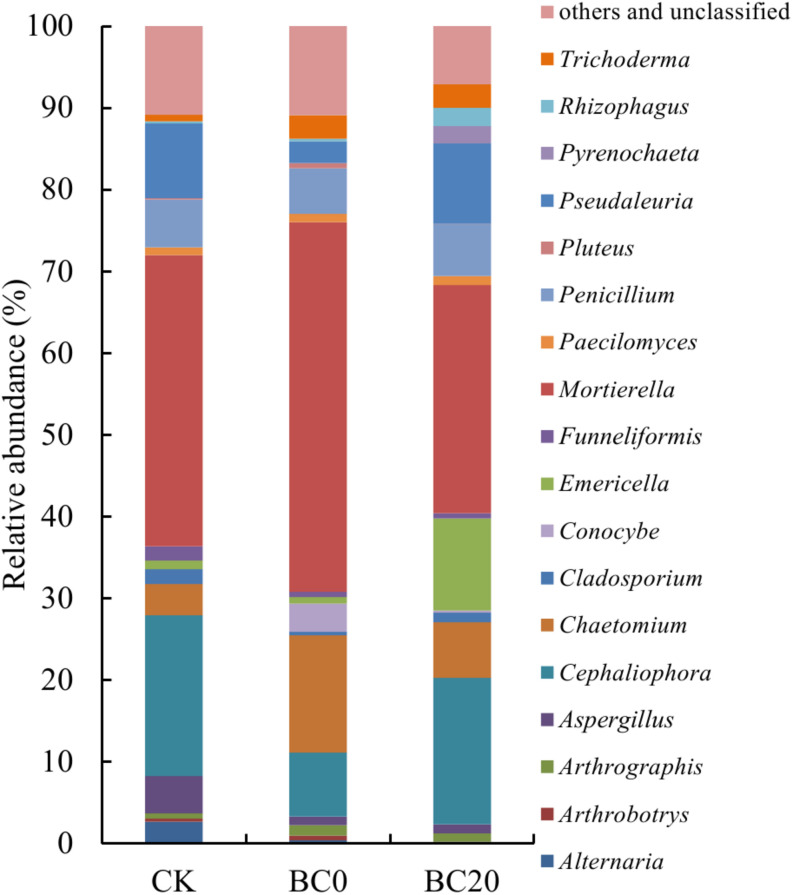
The relative abundance of the top 18 fungal genera in the rhizosphere at 30 days after transplanting.

**FIGURE 5:**
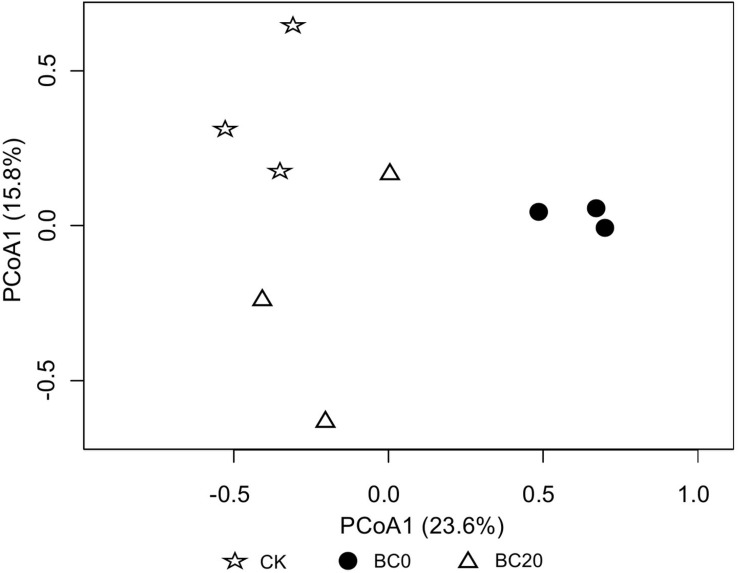
Principal coordinate analysis (PCoA) of fungal communities at 30 days after transplanting.

A comparison of the relative abundance of the different fungal genera with an abundance of >1% revealed significant differences with respect to the different treatments at 30 days after planting (Figure 4). The dominant genera were *Mortierella* (27.94–45.19%), *Cephaliophora* (7.82–19.66%), *Chaetomium* (3.84–14.35%), *Pseudaleuria* (2.67–9.12%), and *Penicillium* (5.62–6.30%), followed by several other genera including *Emericella*, *Aspergillus*, *Trichoderma*, etc. PCoA clearly revealed that biochar amendment shifted the rhizosphere fungal community. In addition, the fungal community of BC0 was clearly separated from that of BC20 (Figure 5).

Groups 1 and 2 in Table 2 included the fungal genera that were higher and lower in relative abundance, respectively, in response to the biochar treatments. Significantly higher relative abundance of *Chaetomium* and *Trichoderma* and significantly lower abundance of *Alternaria* and *Cladosporium* were observed under the biochar treatments than under CK. Biochar treatments also enriched the genera *Conocybe*, *Paecilomyces*, and *Arthrographis* and depleted *Cephaliophora* and *Aspergillu*s. In addition, BC0 and BC20 had different effects on several fungal genera, as observed for group 3. BC0 significantly increased the relative abundance of *Mortierella* by 17.25% and reduced those of *Pseudaleuria*, *Emericella*, and *Pyrenochaetopsis* by 7.16, 10.45, and 2.09%, respectively, compared with BC20.

**TABLE 2:** Fungal genera (average relative abundance > 1%) significantly changed by biochar amendment at 30 days after transplanting.

		CK	BC0	BC20
Group 1	*Chaetomium*	3.84 ± 1.26b	14.35 ± 3.31a	6.82 ± 4.42b
	*Trichoderma*	0.78 ± 0.53b	2.86 ± 0.41a	2.88 ± 0.93a
	*Conocybe*	0a	3.42 ± 2.93a	0.26 ± 0.15a
	*Paecilomyces*	0.94 ± 0.28a	1.02 ± 0.14a	1.10 ± 0.34a
	*Arthrographis*	0.65 ± 0.25a	1.28 ± 0.43a	0.99 ± 0.47a
Group 2	*Cephaliophora*	19.66 ± 13.08a	7.82 ± 0.79a	17.91 ± 4.99a
	*Aspergillus*	4.54 ± 3.34a	1.02 ± 0.63a	1.12 ± 0.30a
	*Cladosporium*	1.83 ± 0.32a	0.44 ± 0.16b	1.15 ± 1.02ab
	*Alternaria*	2.69 ± 1.34a	0.42 ± 0.32b	0.10 ± 0.08b
Group 3	*Mortierella*	35.59 ± 6.59ab	45.19 ± 4.36a	27.94 ± 4.36b
	*Pseudaleuria*	9.12 ± 3.30a	2.67 ± 1.23b	9.83 ± 1.88a
	*Emericella*	1.07 ± 0.18b	0.84 ± 0.35b	11.29 ± 5.46a
	*Pyrenochaetopsis*	0b	0b	2.09 ± 1.62a

*Different letters after the values in the same row indicate significant differences (p < 0.05) according to one-way ANOVA (n = 3). Group 1 includes the fungal genera with higher relative abundances under the biochar treatments. Group 2 includes fungal genera with lower relative abundances under the biochar treatments. Group 3 includes fungal genera that responded differently to the biochar treatments.*

#### Fungal Abundance

**FIGURE 6:**
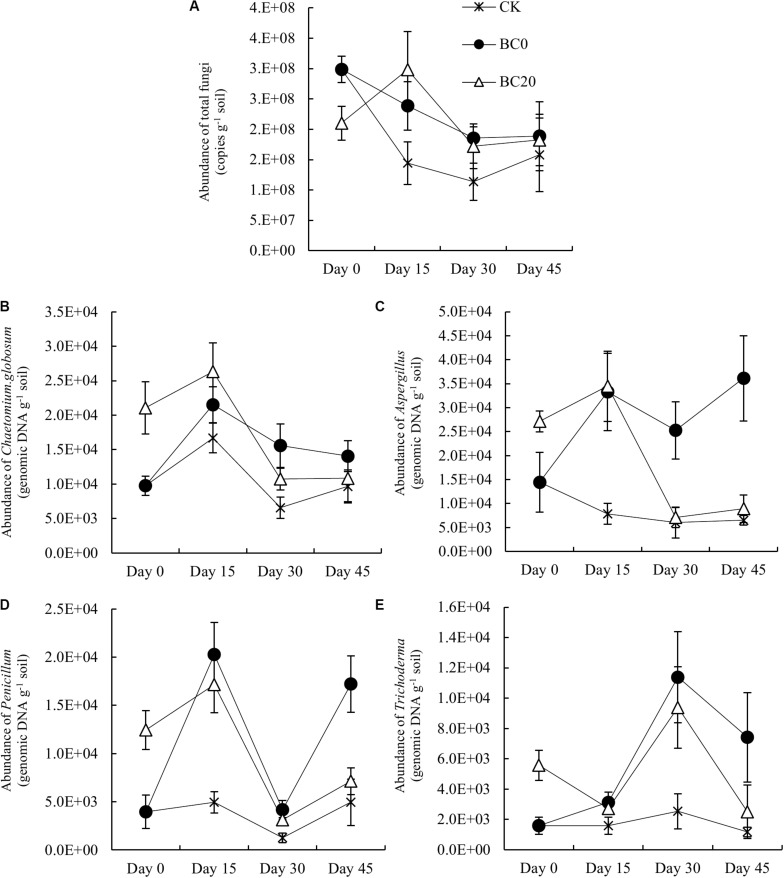
Temporal effects of biochar applied at different times on the abundance of total rhizosphere fungi **(A)** and potential rhizosphere-associated biocontrol fungi during the growing period. The biocontrol fungi were *Chaetomium globosum* **(B)**, *Aspergillus* **(C)**, *Penicillium* **(D)**, and *Trichoderma* **(E)**. *Bars* represent average values ± standard errors (*n* = 3).

The qPCR results showed that the fungal abundance increased significantly under BC20 compared with that under CK after pepper planting (Figure 6A). BC0 resulted in a consistently higher fungal abundance during the entire growing period than CK. Fungal abundance under BC0 was higher than that under BC20 at 30 and 45 days after planting, but the differences were not significant. In addition, the differences in fungal abundance between the CK and biochar treatments gradually decreased, suggesting a diminishing biochar-mediated proliferative effect on rhizosphere fungi over time.

Figures 6B–E indicate that the abundance of *C. globosum*, *Aspergillus*, and *Penicillium* under BC20 subsequently increased slightly and then deceased rapidly, but remained higher than that under CK. BC0 showed a strong enrichment of these biocontrol fungi during the entire growing period. The biocontrol fungal abundance under BC0 was higher or significantly higher than under BC20 at 30 and 45 days after planting. Compared to CK, the abundance of *C. globosum*, *Aspergillus*, *Penicillium*, and *Trichoderma* increased 0.46-, 4.56-, 2.48-, and 5.37-fold, respectively, under BC0 and by 1.14-, 0.12-, 0.37-, and 0.44-fold, respectively, under BC20 at 45 days after planting. Thus, the shorter the duration of biochar in the soil, the stronger the increase in the abundance of biocontrol fungi it induced.

#### Correlation Analyses of Fungal Abundance and Disease Severity

Table 3 shows that the abundance of *P. capsici* correlated positively (*p* < 0.01) with the disease index. The abundance of *P. capsic*i correlated negatively (*p* < 0.01) with the abundance of *C. globosum*, *Aspergillus*, and *Penicillium* as well as with *Trichoderma* at *p* > 0.05. Similarly, disease severity correlated negatively (*p* < 0.01) with the abundance of *C. globosum*, *Aspergillus*, and *Penicillium* as well as with the abundance of *Trichoderma* at *p* < 0.05. In addition, the total fungal abundance correlated negatively with both the disease index and abundance of *P. capsici* at *p* < 0.05. These correlations suggested that the abundance of total fungi, *C. globosum*, *Aspergillus*, *Penicillium*, and *Trichoderma* may be the important factor that suppresses the pathogen and controls Phytophthora blight of pepper in response to biochar amendment.

**TABLE 3:** Relationship of disease severity and pathogen abundance to fungal abundance based on correlation analysis of multi-time point data (15, 30, and 45 days after transplanting).

	Disease severity	*Phytophthora capsici*
*P. capsici*	0.532**	–
Total fungi	−0.441*	−0.425*
*C. globosum*	−0.759**	−0.667**
*Aspergillus*	−0.707**	−0.518**
*Penicillium*	−0.597**	−0.621**
*Trichoderma*	−0.446*	−0.307

*Significant at p < 0.05; **Highly significant at p < 0.01.

### Biocontrol Efficacy of Biochar-Enriched Biocontrol Fungi

Two *Aspergillus* strains, three *Chaetomium* strains, three *Penicillium* strains, and three *Trichoderma* strains enriched by BC0 were screened by comparing their colony characteristics on PDA plates from CK and BC0. The results of the molecular identification are shown in Supplementary Table S2. Two *Aspergillus* (AS1 and AS2), two *Chaetomium* (CH1 and CH3), three *Penicillium* (PE1, PE2, and PE3), and two *Trichoderma* (TR1 and TR3) strains were confirmed to be enriched in response to biochar amendment according to their colonizations in the rhizospheres with and without biochar amendment (data not shown).

In the pot experiment, nine antagonistic fungi showed major variations in the reduction of disease severity (Table 4). At 15 days after planting, *Penicillium* PE1 had no control effect. The control efficacies of the other eight fungal strains were in the range of 18.27–59.62%. In particular, *Aspergillus* AS1 had the highest control efficacy of 59.62%, followed by 51.92% and 45.19% for *Trichoderma* TR3 and TR1, respectively, and 44.23% for *Chaetomium* CH1. The disease indices of all treatments increased with extended planting time, while the control efficacies of the biocontrol strains continued to decline. At 30 days after planting, the control efficacy of *Trichoderma* TR3 was 30.53%, representing the best biocontrol strain, followed by 24.43% for *Aspergillus* AS1. In addition, the control efficacies of two *Chaetomium* strains were 17.37 and 19.08%, while of the *Penicillium* strains, only PE3 had a control efficacy of 15.27%. In short, the *Aspergillus* and *Trichoderma* strains had the best control effect, followed by the *Chaetomium* strains, and then the *Penicillium* strains.

**TABLE 4:** Disease severity, pathogen abundance, and control efficacy of beneficial fungi in *Phytophthora capsici*-infested pepper seedlings.

Treatment	15 days after transplanting			30 days after transplanting		
	Disease severity (%)	Pathogen abundance (10^5^ copies g^–^^1^)	Control efficacy (%)	Disease severity (%)	Pathogen abundance (10^5^ copies g^–^^1^)	Control efficacy (%)
PC	36.11 ± 6.67*ab*	16.53 ± 1.53^a^	–	90.97 ± 4.34*ab*	20.81 ± 3.73^a^	–
PC + AS1	14.58 ± 6.98^d^	6.13 ± 0.41^d^	59.62	68.75 ± 10.42^c^	12.91 ± 1.33*bcd*	24.43
PC + AS2	20.83 ± 6.38*cd*	5.27 ± 0.36^d^	42.31	71.53 ± 8.67^c^	8.12 ± 1.07^e^	21.37
PC + CH1	20.14 ± 5.34*cd*	8.60 ± 1.29^c^	44.23	73.61 ± 2.41^c^	15.36 ± 2.30^b^	19.08
PC + CH3	23.96 ± 4.57*cd*	9.74 ± 1.90*bc*	33.65	71.53 ± 11.47^c^	13.78 ± 1.63*bc*	17.37
PC + PE1	41.67 ± 7.60^a^	11.26 ± 0.97^b^	–15.39	92.36 ± 6.01^a^	20.69 ± 2.94^a^	–1.53
PC + PE2	26.04 ± 5.97*bcd*	12.09 ± 2.65^b^	27.88	88.89 ± 1.20*ab*	20.02 ± 1.82^a^	2.29
PC + PE3	29.51 ± 7.39*bc*	9.93 ± 0.84*bc*	18.27	77.08 ± 2.08*bc*	15.15 ± 1.42^b^	15.27
PC + TR1	19.79 ± 4.84*cd*	4.47 ± 0.66^d^	45.19	72.22 ± 10.69^c^	9.13 ± 1.36*de*	20.61
PC + TR3	17.36 ± 3.29^d^	3.82 ± 0.75^d^	51.92	63.19 ± 11.47^c^	10.19 ± 2.54*cde*	30.53

*Different letters after the values in the same column indicate significant differences (p < 0.05) according to one-way ANOVA (n = 3). Aspergillus strains: AS1 and AS2. Chaetomium strains: CH1 and CH3. Penicillium strains: PE1, PE2, and PE3. Trichoderma strains: TR1 and TR3. PC, soil not inoculated with biocontrol bacteria.*

The abundance of *P. capsici* and the disease indices of all treatments showed the same trend, i.e., a high disease index indicated high pathogen abundance. All seven biocontrol strains (except PE1 and PE3), which showed a control effect, were associated with a significantly lower abundance of *P. capsici* than the control. The pathogen abundance among the seven treatments with similar control effects did not differ significantly.

## Discussion

Biochar demonstrated its possibility as a control agent against Phytophthora blight of pepper, supporting similar control effects of soilborne diseases observed in previous studies (Jaiswal et al., 2017; Zhang et al., 2017; Gao et al., 2019; Jaiswal et al., 2019; Chen et al., 2020). Furthermore, biochar applied before planting showed a significantly higher control effect than that applied 20 days before planting, indicating its declining control effect with prolonged application time prior to pepper planting. We assessed the chemical properties, fungal community composition, and abundance of potential biocontrol fungi in the rhizosphere soil, providing novel insights into the mechanisms underlying the biochar-mediated control of soilborne diseases.

Supporting previous studies (Gul et al., 2015; Yao et al., 2017; Zhang et al., 2017; Chen et al., 2020), biochar amendment directly affected the soil chemical properties, in particular soil pH, EC, and the contents of organic matter, available P, and available K. Elemental P and K can promote sugar and protein metabolism in plants, stimulate root growth, accelerate root absorption, and effectively alleviate root diseases (Gupta et al., 2017). Zhang et al. (2017) and Chen et al. (2020) reported that biochar-induced changes in the soil chemical properties were favorable for beneficial microorganisms, but unfavorable for *Ralstonia* and bacterial wilt. Similarly, in our study, the increases in pH, EC, and contents of available P and available K caused by biochar amendment were conducive to increasing the relative abundance of beneficial fungi, such as *Trichoderma*, *Chaetomium*, *Penicillium*, *Emericella*, and *Paecilomyces* (Supplementary Figure S2). In addition, the soil EC and the contents of organic matter, available P, and available K correlated significantly and negatively with the abundance of pathogens and disease severity (Supplementary Table S3), indicating a significant association between the soil chemical properties and disease suppression in response to biochar amendment. As there was little difference between BC0 and BC20 in terms of soil chemical properties, the difference in the disease control effect between these two biochar treatments was most likely due to the differences in the microbial properties within the rhizosphere.

Fungal abundance significantly increased in the biochar-amended soils, which was in agreement with the results of Bamminger et al. (2014) and Yao et al. (2017). Although this stimulation was weakened with extended planting time, fungal abundance in the biochar-amended soils was still greater than that in unamended soils during the growing period. Zhang et al. (2017), Jaiswal et al. (2018), and Li et al. (2019) indicated that biochar amendment positively influences fungal richness and diversity. Supporting that, significantly higher fungal richness and diversity indices were found in the biochar-amended soils. Many studies have documented that soil microbial richness and diversity and the ratio of pathogens to fungi may contribute to soil disease suppression (Bender et al., 2016; Fra̧c et al., 2018; Huang et al., 2018; Saleem et al., 2019), suggesting that the lower disease severity in the biochar treatments could be partly linked to the increased fungal abundance as well as fungal richness and diversity. However, the difference between BC0 and BC20 was small, indicating that the significant difference in disease control between the two biochar treatments was mostly due to the differences in the fungal community composition.

High-throughput sequencing revealed that the relative abundance of certain fungal genera significantly decreased or increased in response to biochar amendment. The relative abundance of *Chaetomium*, *Paecilomyces*, *Penicillium*, and *Trichoderma* was higher in the biochar-amended soils. These four genera have been reported to be linked to the promotion of plant growth, production of antibiotic compounds, induction of plant defenses, and suppression of soilborne disease (Howell, 2003; André and Schmoll, 2010; Sibounnavong et al., 2011; Khan et al., 2012; Prakob et al., 2012; Bladt et al., 2013; Shanthiyaa et al., 2013; Saldajeno et al., 2014). Another important finding was that the relative abundance of *Alternaria* and *Cephaliophora*, which may cause plant disease (Sweta et al., 2014; Mukesh et al., 2017), decreased under the biochar treatments, suggesting that biochar could suppress crop pathogens. The differences noted between the BC0 and BC20 treatments, such as a higher abundance of *Chaetomium* and a lower abundance of *Cephaliophora*, suggest that the BC0-induced fungal community may have a higher soilborne disease suppression ability.

Consistent with the high-throughput sequencing results, the qPCR results showed that the abundance of *C. globosum*, *Penicillium*, and *Trichoderma* significantly increased in the biochar-amended soils. However, the higher abundance of *Aspergillus* in the biochar treatments was in contrast to the results of the high-throughput sequencing. This may be related to the accuracy and adaptability of the two methods (Murray et al., 2011). The increased abundance of *Aspergillus*, *Penicillium*, and *Trichoderma* was probably related to the degradation of polycyclic aromatic hydrocarbons in the biochar *via* the production of enzymes (Anyika et al., 2015; Al-Hawash et al., 2018), which was in agreement with other studies (Jaiswal et al., 2017, 2018; Vecstaudza et al., 2018). Several authors have reported that *C. globosum*, *Aspergillus*, *Penicillium*, and *Trichoderma* could control plant diseases caused by *Phytophthora* spp. and suppress these pathogens (Fang and Tsao, 1995; Kang et al., 2005; Shanthiyaa et al., 2013; Widmer, 2014). Correlation analyses indicated that the biochar-mediated disease suppression was closely associated with the proliferation of *C. globosum*, *Aspergillus*, *Penicillium*, and *Trichoderma*. The biochar-mediated proliferation of biocontrol fungi may contribute to reducing the abundance of *P. capsici* due to the production of antibiotic compounds with activities against pathogens (Park et al., 2005; André and Schmoll, 2010; Bladt et al., 2013). The *Aspergillus*, *Chaetomium*, and *Trichoderma* strains enriched by the biochar exhibited strong suppression of *P. capsici* and Phytophthora blight of pepper. However, the *Penicillium* strains, except one, showed little control efficacy and failed to reduce the disease. Thus, the enrichment of *Aspergillus*, *Chaetomium*, and *Trichoderma* probably contributed much to the disease suppression effects of biochar.

We assumed that soil incubated with biochar for 20 days could form a soil microbial community that is adverse to soilborne pathogens, thereby improving the soil disease suppression. However, although the abundance of potential biocontrol fungi was high under BC20 before planting, the effect of this treatment on Phytophthora blight of pepper was profoundly reduced compared to that of BC0. The proliferation of pathogens and plant morbidity are time-dependent processes. In the mid- and late growing periods, the abundance of biocontrol fungi under BC0 was markedly increased relative to that under BC20, which is probably an important reason for the higher control effect of BC0.

Overall, our results indicate that biochar-induced improvement in the rhizosphere fungal community, especially the increased abundance of total fungi and beneficial fungi as well as the augmented fungal richness and diversity, conferred inhibition of *P. capsici* and Phytophthora blight of pepper. Biochar-mediated improvement of soil chemical properties had positive effects on beneficial soil fungi and disease suppression. The disease control effect of biochar was significantly weakened with a prolonged period between the application and planting, which may be largely explained by the short-term promoting effect of biochar amendment on the abundance of biocontrol fungi, such as *Aspergillus*, *Chaetomium*, and *Trichoderma*. Further work is required to pinpoint more biochar-enriched microorganisms that contribute to disease suppression and to elucidate the contribution of each of these microorganisms on controlling soilborne disease.

## Data Availability Statement

The original DNA sequence data were deposited in the National Center for Biotechnology Information (NCBI) with accession number SRP224915.

## Author Contributions

GW, YM, and RG designed the study. GW performed the experiments and was involved with writing the manuscript. YM, RG, and HC contributed to revising the manuscript. JL participated in the greenhouse and lab work. RG helped with sequence data analysis. All authors contributed to the article and approved the submitted version.

## Conflict of Interest

The authors declare that the research was conducted in the absence of any commercial or financial relationships that could be construed as a potential conflict of interest.
