# Supplementary material for: Biochar-Mediated Control of Phytophthora Blight of Pepper Is Closely Related to the Improvement of the Rhizosphere Fungal Community
Source: Front Microbiol. 2020 Jul 8;11:1427. doi: 10.3389/fmicb.2020.01427 (PMC7360685; doi:10.3389/fmicb.2020.01427)
Supplement: Supplementary file 1 [file Table_1.DOCX]

Figure S1. Effect of biochar amendment on fungal community structure after incubation for 20 days. Treatments included CK (soil not amended with biochar) and BC20 (soil amended with biochar 20 days before transplanting).


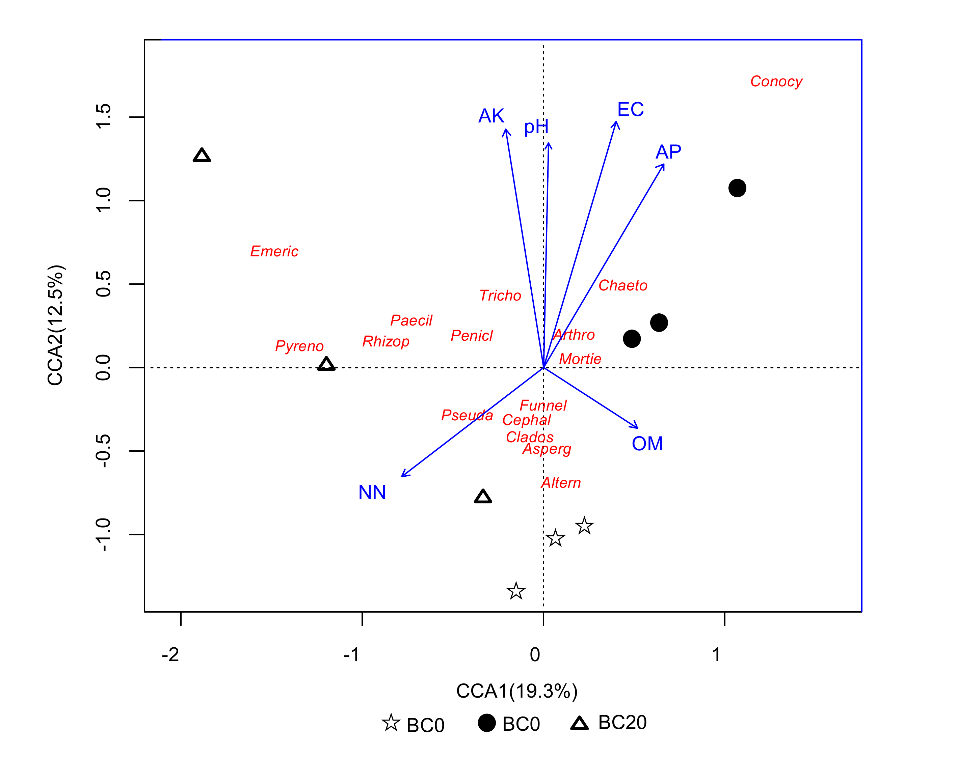


Figure S2. Canonical correspondence analysis (CCA) of the fungal community composition associated with soil chemical properties at 30 days after transplanting. EC, electrical conductivity; OM, Organic matter; NN, nitrate N; AP, available P; AK, available K; Altern, *Alternaria*; Arthro, *Arthrographis*; Asperg, *Aspergillus*; Cephal*, Cephaliophora;* Chaeto*, Chaetomium;* Clados*, Cladosporium;* Conocy*, Conocybe;* Emeric*, Emericella*; Funnel, *Funneliformis*; Mortie, *Mortierella*; Paecil, *Paecilomyces*; Penici, *Penicillium*; Pseuda, *Pseudaleuria*; Pyreno, *Pyrenochaeta*; Rhizop, *Rhizophagus*; and Tricho, *Trichoderma.* Treatments included CK (soil not amended with biochar), BC0 (soil amended with biochar just before transplanting) and BC20 (soil amended with biochar 20 days before transplanting).

Table S1. Effect of biochar amendment on soil chemical properties after incubation for 20 days

|  | CK | BC20 |
| --- | --- | --- |
| pH | 7.44±0.09a | 7.52±0.08a |
| EC (μs cm^-1^) | 1849.2±28.3b | 2300.5±55.2a |
| Organic matter (g kg^-1^) | 29.4±2.6b | 33.4±2.0 a |
| Nitrate N (mg kg^-1^) | 116.7±4.9a | 118.9±2.7a |
| Available P (mg kg^-1^) | 123.6±5.6b | 150.1±3.9a |
| Available K (mg kg^-1^) | 144.5±7.1b | 536.3±14.2a |

Different letters after the values of the same row indicate significant differences (p < 0.05) according to one-way ANOVA (n=3). Treatments included CK (soil not amended with biochar) and BC20 (soil amended with biochar 20 days before transplanting).

Table S2. Identification of fungal isolates based on BLAST analysis of ITS sequences

| Species |  | The strains which have the highest identity from NCBI | Homology  ( %) |
| --- | --- | --- | --- |
| *Aspergillus* | AS1 | *Aspergillus aculeatus* strain NFML_CH59_131 | 100 |
|  | AS2 | *Aspergillus niger* strain MSR3 | 99 |
| *Chaetomium* | CH1 | *Chaetomium* sp. strain LZZ0017 | 100 |
|  | CH2 | *Chaetomium* sp. BMP3044 | 99 |
|  | CH3 | *Chaetomium globosum* isolate UOM AB | 99 |
| *Penicillium* | PE1 | *Penicillium janthinellum* strain CWG3 | 99 |
|  | PE2 | *Penicillium citrinum* isolate M27 | 99 |
|  | PE3 | *Penicillium menonorum* NRRL 50410 | 99 |
| *Trichoderma* | TR1 | *Trichoderma* sp. BAB-4829 | 100 |
|  | TR2 | *Trichoderma asperellum* strain RHg | 99 |
|  | TR3 | *Trichoderma longibrachiatum* strain IHB F 539 | 99 |

Table S3. Relationships of disease severity and pathogen abundance with the soil chemical properties based on correlation analysis of multitime point data (15, 30, and 45 days after transplanting).

|  | Disease severity | *P. capsici* |
| --- | --- | --- |
| pH | 0.094 | -0.003 |
| EC | -0.881^**^ | -0.480^*^ |
| Organic Matter | -0.417^*^ | -0.245 |
| Nitrate N | -0.507^*^ | -0.334 |
| Available P | -0.720^**^ | -0.311 |
| Available K | -0.712^**^ | -0.402^*^ |

* Significant at p < 0.05; **Highly significant at p < 0.01.
